# Prediction of Placental Barrier Permeability: A Model Based on Partial Least Squares Variable Selection Procedure

**DOI:** 10.3390/molecules20058270

**Published:** 2015-05-07

**Authors:** Yong-Hong Zhang, Zhi-Ning Xia, Li Yan, Shu-Shen Liu

**Affiliations:** 1 Medicine Engineering Research Center, School of Pharmacy, Chongqing Medical University, Chongqing 400016, China; 2 College of Chemistry and Chemical Engineering, Chongqing University, Chongqing 400030, China; E-Mail: znxia@cqu.edu.cn; 3 Department of Chinese Traditional Medicine, Chongqing Medical University, Chongqing 400016, China; E-Mail: ly200802958@163.com; 4 State Key Laboratory of Pollution Control and Resources Reuse, Key Laboratory of Yangtze River Water Environment, Ministry of Education, College of Environmental Science and Engineering, Tongji University, Shanghai 200092, China

**Keywords:** placental barrier permeability, descriptors based on Dragon software, PLS regression, variable importance in projection (VIP), validation, application domain

## Abstract

Assessing the human placental barrier permeability of drugs is very important to guarantee drug safety during pregnancy. Quantitative structure–activity relationship (QSAR) method was used as an effective assessing tool for the placental transfer study of drugs, while *in vitro* human placental perfusion is the most widely used method. In this study, the partial least squares (PLS) variable selection and modeling procedure was used to pick out optimal descriptors from a pool of 620 descriptors of 65 compounds and to simultaneously develop a QSAR model between the descriptors and the placental barrier permeability expressed by the clearance indices (CI). The model was subjected to internal validation by cross-validation and *y*-randomization and to external validation by predicting CI values of 19 compounds. It was shown that the model developed is robust and has a good predictive potential (*r*^2^ = 0.9064, *RMSE* = 0.09, *q*^2^ = 0.7323, *r_p_*^2^ = 0.7656, *RMSP* = 0.14). The mechanistic interpretation of the final model was given by the high variable importance in projection values of descriptors. Using PLS procedure, we can rapidly and effectively select optimal descriptors and thus construct a model with good stability and predictability. This analysis can provide an effective tool for the high-throughput screening of the placental barrier permeability of drugs.

## 1. Introduction

More and more prescription and non-prescription drugs are directly used in pregnant women, which will cause fetuses exposed to the drugs from the mother transferring across the placental barrier [1,2,3,4]. In the past decades, women who took one drug during pregnancy has accounted for 90% and at least 10 drugs for 4% in China, according to the data of the Ministry of Health, while pregnant women consumed an average of 2.3 drugs in North America [5,6]. These drugs might cause fetal toxicity or teratogenicity but do not hurt the mother. As the pregnancy rates in women over the age of 40 have been continually growing [7], from now on, the mean of drugs consumed in pregnancy is expected to increase. Therefore, to guarantee drug safety during pregnancy, the urgent demand for accurate fetal health risk assessment has led to the development of *in vitro* and *in vivo* experimental models to research the human placental barrier permeability of drugs.

Human placenta is a unique organ for feto-placental-maternal circulation in pregnancy [8,9]. Although animal experiments were applied to evaluate the placental barrier permeability of drugs, human tissue and cells are still the best choice [10,11]. However, the *in vivo* risk assessment studies of exposures to drugs are forbidden in humans due to ethical reasons. To avoid the ethical problems, several *in vitro* models emerged, including primary trophoblastic cells, immortal cell lines of placental origin and explants as human placental perfusion [10,12,13]. However, these *in vitro* experiments are time-consuming and demanding methods.

Quantitative structure activity-property relationship (QSAR/QSPR) study has been extensively used to develop a model between the chemical structures of molecules and the available biological properties, and to predict the properties which must be obtained through *in vivo* or *in vitro* experiments [14,15,16,17]. For example, the QSAR technique has been used in the drug ADME/T assessment [18,19,20,21,22,23,24,25,26]. However, there is only a little literature on QSAR of placental barrier permeability. Hewitt *et al.* [27] established five different QSAR models, but all of them just carried out internal validating, not did external validation. Giaginis *et al*. [28] created a Partial Least Squares (PLS) regression model by the original 16 variables using Multivariate Data Analysis (MVDA). The model had lower *r*^2^ and bigger *RMSE* (in training set: *r*^2^ = 0.72, *q*^2^ = 0.69, *RMSE* = 0.16 and in test set: *RMSP* = 0.16). Meanwhile, the model was not defined with an appropriate application domain.

It is well known there are two key steps in QSAR. One is creating the molecule descriptor, the other is modeling. As pointed out in our previous work [29], the first step is very easy because a great deal of descriptors can be rapidly acquired using software such as MOE [30] and DRAGON [31]. At present, there are many QSAR modeling techniques such as multiple linear regression (MLR), support vector machine (SVM), principal component analysis (PCA) and PLS regression [32,33,34]. However, how to select the descriptors closely related to a required biological property from a big descriptor pool in order to establish a robust and predictable model is becoming a bottleneck problem. In the review written by Gonzalez *et al*. [35], different variable selection methods were discussed, including stepwise-regression, optimal subset, genetic algorithm (GA) and artificial neural network (ANN). MVDA [28] is commonly applied as a powerful conventional statistical tool for variable selection. However, many of the variable selection methods mentioned above have some defects. For example, the stepwise regression, optimal subset and conventional statistical method are relatively appropriate only for a few variables. In addition, ANN as a non-linear method creates difficulties in interpretation. Furthermore, the most well-known advantage of GA is in establishing a robust model, but the GA results very much depend on the number of generations allowed to evolve. Luckily, the PLS is a linear, numerous variable, and non-random variable selection and modeling method. PLS cannot only avoid collinearity or auto-correlation problems but also address the puzzles in ANN and GA. Therefore, PLS as a rapid and effective method was widely used to develop robust and predictable QSAR models [36,37,38,39].

In this study, the molecular descriptors are computed using DRAGON software and the PLS procedure [29,40] is chosen to select optimal descriptors and develop a QSAR model between the placental barrier permeability expressed by CI and the optimal descriptors. At the same time, the PLS regression model is subject to rigid internal and external validation and the optimal variables with high VIP values are rationally illustrated.

## 2. Results and Discussion

### 2.1. PLS Variable Selection

The selection of optimal variables is performed step by step. When *A* = 8, the VIP values of 396 descriptors are greater than 1.000 in the PLS model based on 620 original descriptors. Then, the 396 descriptors with high VIPs act as new original ones and a new PLS model is developed in the same way. When *A* = 7, the VIP values of 286 descriptors are greater than 1.00 in the new PLS model. Again, when *A* = 8235, descriptors have high VIP values. Relevant statistical results in PLS variable selection were shown in Table 1.

**Table 1:** The statistical results of variable selection by PLS method.

*m*	*A*	*r^2^*	*RMSE*	*q^2^*	*RMSV*
620	8	0.9801	0.04	0.3715	0.25
396	7	0.9716	0.05	0.5569	0.20
286	8	0.9745	0.05	0.6532	0.18
235	8	0.9751	0.05	0.6773	0.17
195	7	0.9573	0.06	0.6984	0.16
163	7	0.9651	0.06	0.7445	0.15
137	7	0.9518	0.07	0.7153	0.16
115	7	0.9368	0.07	0.6941	0.17
100	7	0.9264	0.08	0.6831	0.17
85	7	0.9302	0.08	0.7125	0.16
79	7	0.9341	0.08	0.7560	0.15
73	7	0.9258	0.08	0.7330	0.15
67	7	0.9169	0.09	0.7022	0.16
62	7	0.9138	0.09	0.7271	0.16
58	7	0.9110	0.09	0.7208	0.16
55	7	0.9115	0.09	0.7303	0.15
48	7	0.9064	0.09	0.7323	0.15
42	7	0.8525	0.11	0.6655	0.17
39	5	0.8115	0.13	0.6350	0.18
34	5	0.7845	0.14	0.6138	0.19

From Table 1, when the number of selected variables, *m* = 163, 79, 62 and 48, the *q*^2^ values of the relevant models have maximum values. For example, when *m* = 55, *q*^2^ = 0.7303; *m* = 48, *q*^2^ = 0.7323; and *m* = 42, *q*^2^ = 0.6655, it means that the value of *q*^2^ was a peak at *m* = 48. Taking into account the number of samples is only 87 compounds, the fewer the number of variables, the better the model. Thus, 48 descriptors are chosen in the final PLS model. Categories and specific names of these 48 optimal descriptors are shown in Table 2.

**Table 2:** The names and types of selected 48 optimal descriptors.

Type of Descriptor	*m*	Name of Descriptor
Constitutional indices	4	Me, O%, nO, nHet
Topological indices	3	DELS, DECC, Psi_i_A
Connectivity indices	1	X0Av
Information indices	3	SIC1,AAC, IC1
2D matrix-based descriptor	5	TI2_L, SM5_X, Chi_Dz(p), SM1_Dz(p), SM6_B(s)
2D autocorrelations	11	MATS3v, GATS1e, ATSC2s, MATS1e, ATSC3e, ATSC1e, ATSC1s, ATSC3s, MATS8i, GATS3v, GATS1s
Burden eigenvalues	1	SpMax3_Bh(s)
P-VS-like descriptors	2	P_VSA_p_2, P_VSA_s_6
Edge adjacency indices	4	Eig03_EA(dm), Eig05_EA(dm), Eig06_EA(dm), SpMAD_B(s)
Functional group counts	3	nRNH2, nHDon, nPyrimidines
Atom-centred fragments	1	O-057
CAST 2D	5	CATS2D_07_DD, CATS2D_04_DD, CATS2D_08_DA CATS2D_05_AP, CATS2D_04_LL
2D atom pairs	2	T(O..O), F05[O-O]
Molecular properties	2	MLOGP, SAdon
Drug-like indices	1	LLS_01

### 2.2. PLS Regression Model

The model between the CIs and 48 descriptors of 65 training set samples was built by PLS regression. The corresponding experimental and calculated CI values of 65 compounds were summarized in Table 3.

**Table 3:** Eighty-eight compounds and their CI observed and calculated values where the compounds with an asterisk (*) refer to ones in the test set.

No.	Name	CI-Obs.	CI-Cal.	No.	Name	CI-Obs.	CI-Cal.
1 *	Abacavir	0.47	0.62	45	Mefloquine	1.57	
2	Acipimox	0.25	0.38	46	Meropenem	0.08	0.16
3 *	Acyclovir	0.17	0.09	47	Metaclopramide	0.40	0.65
4 *	Alanine	0.30	0.40	48	Metformin	0.34	0.44
5	Alfentanil	0.75	0.68	49	Methadone	0.83	0.97
6	PAH	0.47	0.41	50 *	Mezlocilline	0.14	–0.08
7 *	Amprenavir	0.38	0.39	51 *	Morphine	0.63	0.36
8 *	Azidothymidine	0.29	0.15	52	Naloxone	0.64	0.46
9	Betamethasone	0.41	0.44	53 *	Nicotine	0.93	0.54
10	Biotin	0.35	0.43	54	Oseltamivir	0.13	0.28
11	Bisheteroypiperazine	0.72	0.65	55	Hydroxyphenytoin	0.52	0.51
12	Buprenorphine	0.29	0.32	56	PCB-52	0.74	0.62
13	Cefoperazone	0.04	0.06	57	Pentamidine	0.04	0.04
14	Cefpirome	0.20	0.02	58	Phenobarbitone	0.52	0.63
15 *	Ceftizoxime	0.12	0.04	59 *	Prednisolone	0.38	0.46
16 *	Chloroprocaine	0.83	0.69	60	Propofol	0.51	0.58
17	L-Leucine	0.62	0.55	61	Pyridoxal	0.37	0.40
18	Lidocaine	0.91	0.96	62	Pyridoxal 5'-phosphate	0.07	0.06
19 *	Bupivacaine	0.73	0.91	63	Pyridoxine	0.56	0.45
20 *	Cimetidine	0.30	0.38	64	Pyrimethamine	1.00	1.03
21	Clavulanic acid	0.06	0.11	65	Quabain	0.07	0.07
22	Cocaethylene	0.78	0.82	66	Ribofl avin	0.69	0.74
23	Cocaine	0.88	0.74	67	Rifabutin	0.37	0.42
24 *	Cortisol	0.50	0.54	68 *	Rifampin	0.12	0.76
25	Cortisone	0.74	0.63	69	Ritodrine	0.10	0.04
26	Creatinine	0.31	0.36	70	Ritonavir	0.09	0.07
27	D4T	0.24	0.25	71 *	Ropivacaine	0.75	0.94
28	DDE	0.61	0.68	72	Rosiglitazone	0.20	0.35
29	Dexamethasone	0.37	0.44	73	Salbutamol	0.40	0.30
30	Dichlorobenzene	0.98	0.99	74	Saquinavir	0.05	0.09
31	Diclofenac	0.79	0.68	75 *	*S*-Ketoprofen	0.39	0.91
32 *	Didanosine	0.31	0.29	76	SR49059	0.31	0.33
33	Ethanol	1.07	1.05	77	Sufentanil	0.66	0.65
34	Fenoterol	0.10	0.18	78	Sulindac	0.47	0.60
35	Ganciclovir	0.17	0.08	79	Sulindac sulfide	0.81	0.64
36 *	Glucose	0.26	0.50	80	Theophylline	0.80	0.64
37	Hydralazine	0.61	0.62	81	Thiopental	0.95	0.89
38	Indinavir	0.39	0.34	82	Ticarcillin	0.04	0.14
39 *	Indomethacin	0.72	0.58	83 *	Triameterene	0.85	0.80
40 *	L-Alpha-acetyl-*N*-normethadol	0.80	0.88	84	Trovafl oxacin	0.19	0.23
41	L-Alphacetylmethadol	0.95	0.92	85	Urea	0.32	0.28
42	Lamivudine	0.23	0.19	86	Valproic acid	0.95	0.93
43	Lysine	0.35	0.29	87	Vinblastine	0.31	0.23
44	Lopinavir	0.73	0.60	88	Zalcitabine	0.22	0.34

Some statistics obtained in modeling were given as follows,

*n* = 65, *A* = 7, *m* = 48, *r*^2^ = 0.9064, *RMSE* = 0.09, *F* = 78.86 (modeling)

**Figure 1:**
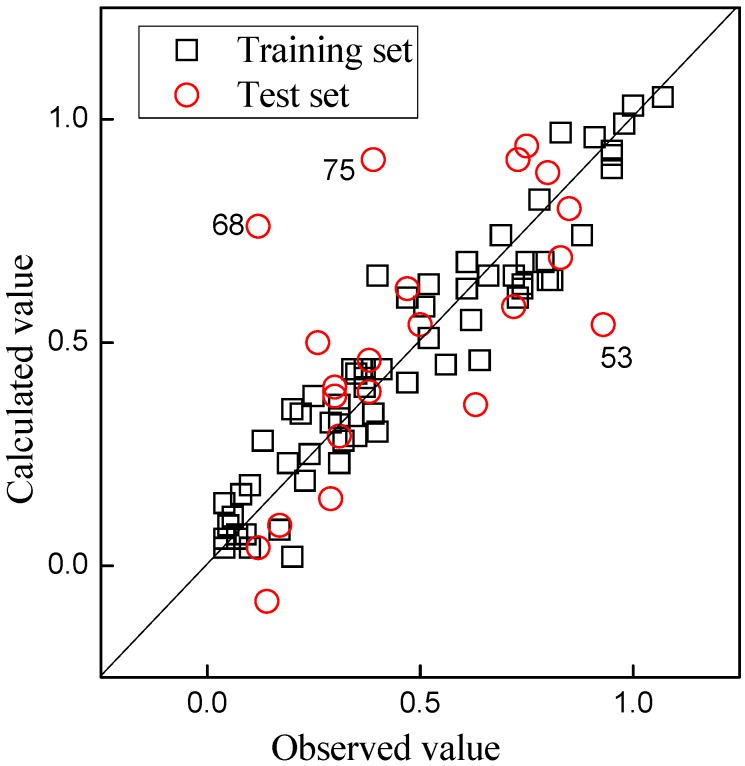
Plot of the CI values calculated by the Partial Least Squares (PLS) models *vs.* those observed.

This model has a good estimation ability (*r*^2^ = 0.9064, *RMSE* = 0.09). The plot of CI values calculated *vs.* those observed was shown in Figure 1. In Figure 1, the little black square is on behalf of the sample in the training set and all squares are evenly and almost symmetrically distributed around the diagonal line, which indicates that the model fit very well and PLS regression model based on 48 optimal descriptors has good estimation ability for the placental barrier permeability of compounds.

### 2.3. Internal and External Validation

In statistical prediction, the following three cross-validation methods are often used to examine a predictor for its effectiveness in practical application: independent dataset test, subsampling test (leave-many-out (LMO) or K-fold cross-validation), and jackknife test (or leave-one-out (LOO) cross-validation) [41]. (i) For the independent dataset test, although all the samples used to test the predictor are outside the training dataset used to train it so as to exclude the “memory” effect or bias, the method for selecting the independent samples to test the predictor could be quite arbitrary unless the number of independent samples is sufficiently large; (ii) For the subsampling test, the concrete procedure usually used in literatures is the five-fold, seven-fold or 10-fold cross-validation. Also, there is another usual procedure named LMO cross-validation. Subsampling covered global sampling in K-fold while there were only small sampling times in LMO cross-validation. The problem with this kind of subsampling test is that the number of possible selections in dividing a benchmark dataset is an astronomical figure even for a very simple dataset, as demonstrated by Equations 28–30 in [42]; (iii) In the jackknife test (or LOO), all the samples in the benchmark dataset will be singled out one-by-one and tested by the predictor trained by the remaining samples. During the process of jackknifing, both the training dataset and testing dataset are actually open, and each sample will be in turn moved between the two. The jackknife test can exclude the “memory” effect. Also, the arbitrariness problem as mentioned above for the independent dataset test and subsampling test can be avoided because the outcome obtained by the jackknife cross-validation is always unique for a given benchmark dataset. Therefore, three test methods were complementary for testifying the QSAR model quality. To reduce the computational time, we adopted the independent testing dataset, LOO and LMO cross-validation in this study.

The above PLS model was internally and externally validated by using the LOO and LMO cross-validation, y-randomization, and predicting the test set samples. It was shown that the model has high stability which is validated by the LOO cross-validation (*q*^2^ = 0.7323, *RMSV* = 0.15). Whether the model is robust or not is still need to do the LMO cross-validation. When M = 2, 3, 4, 5, and 6, respectively, the mean and 95% confidence intervals of the validated *q*^2^_LMO_ values were listed in Table 4. The maximum average value of *q*^2^_LMO_ is (0.6932 ± 0.0148) obtained in the L5O validation, while the minimum one is (0.5441 ± 0.0217) in the L6O. All average *q*^2^_LMO_ values in the LMO cross-validation are bigger than 0.5. Both the results of LOO and LMO cross-validation indicate that the model is very robust.

**Table 4:** The statistical parameters and their values in PLS regression model.

Model Parameter		Value	
*A*		7	
*r*^2^		0.9064	
*RMSE*		0.09	
*q*^2^(*LOO*)		0.7323	
*RMSV*		0.15	
*q*^2^(*L2O*)		0.6620 (±0.0195)	
*q*^2^(*L3O*)		0.6496 (±0.0147)	
*q*^2^(*L4O*)		0.6638 (±0.0169)	
*q*^2^(*L5O*)		0.6932 (±0.0148)	
*q*^2^(*L6O*)		0.5441 (±0.0217)	
*Y-Randomization*	*r*^2^*_Yrand_*	0.3740 (±0.0152)	
	*q*^2^*_Yrand_*	−1.1573 (±0.1952)	
*r_p_*^2^		0.4201(*n_p_* = 22)	0.7656(*n_p_* = 19)
*RMSP*		0.23	0.14

Further internal validation of the model was performed using *y*-randomization (repeated 10 times). The result (*r*^2^*_Yrand_* and *q*^2^*_Yrand_*) obtained from the *y*-randomization is also displayed in Table 4. All of the *q*^2^*_Yrand_* values are following negative values and the value of *r*^2^*_Yrand_* is equal to 0.3740 (±0.0152), and they belong to the area of 0.3 ˂ *r*^2^*_Yrand_* ˂ 0.4, which indicates that the variance of the model is acceptable accidental correlation [43]. Thus, the results of the internal validation indicate the model is still dependable.

The model was externally validated by predicting 22 samples in the test set in order to assess the actual predictive power of the QSAR model. Then, the optimal set of 48 descriptors of 22 compounds in the test set was picked out from 620 descriptors. The PLS regression model was employed to predict the CI values of samples in the test set (*n_p_* = 22, *A* = 7, *m* = 48, *r_p_*^2^ = 0.4201, *RMSP* = 0.23). The calculated CI values of 22 compounds were summarized in Table 3. The plot of CI values calculated *vs.* those observed was also shown in Figure 1 and the red circle is on behalf of the sample in the test set. As shown in Figure 1, there are only three particularly obvious red circles (representing the compounds of nos. 53, 68, and 75) far away from the diagonal line which indicated that the predicted CI values of these three compounds should be doubtful. Although the predicted *r_p_*^2^ (0.4201) and *RMSP* (0.23) are unsatisfactory, the model can be considered to have predictive power when three outliers are taken into account. It is rational because the absolute predictive residuals of the outliers are higher than 3 × *RMSE*, the residual being −0.39 for the compound of no. 53, 0.64 for no. 68, and 0.52 for no. 75 (see Table 3). If these three compounds considered as outliers were deleted from the test set, the model has high predictive potential for the remaining 19 compounds (*r_p_^2^* = 0.7656, *RMSP* = 0.14).

### 2.4. Application Domain

**Figure 2:**
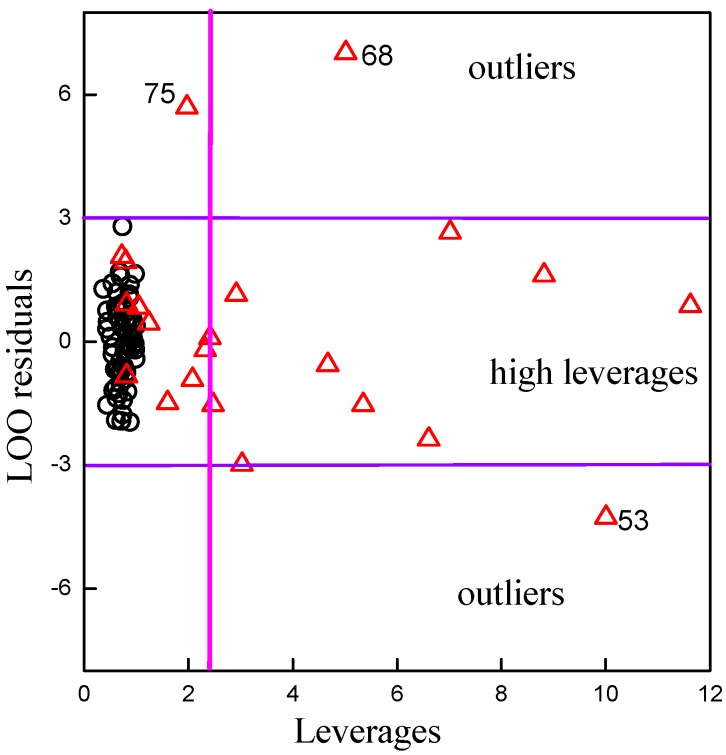
Willam’s maps for model’s application domain. Plot of the LOO standardized residuals *versus* leverage of the PLS model. The small black circles represent samples of the training set, and the red up-triangle on behalf of the samples in test set.

The structure of application domain of the model was defined by leverage [44]. The leverage values are calculated for every compound and plotted *vs*. standard residuals referred to as the Willam’s plot [43,45]. The control leverage h* is fixed at 2.22 (=3 × 48/65). There are many biological and pharmaceutical uncertainties in the animal experiments, which generally cause bigger error in the data. The restrict residual is taken by the empirical value, defined as three times the deviation. The calculated values of the training set and the predicted values of the test set are displayed in the Willam’s plot in Figure 2.

From Figure 2, all black circles (compounds in training set) follow the middle-left region, the best domain with appropriate leverages and residues. Some up-triangles are distributed in the middle-left region, which indicates that the predicted value of these compounds should be proposed. Some other up-triangles are distributed into the middle-right region, which indicates that the predicted CI value can be extrapolated from the model. The leverages of these compounds are high, but the standard residual values of these compounds are less than 3. Therefore, these compounds are still kept in the model, and these predicted results must be used with great care. There are three up-triangles following the up and down regions in Figure 2, which means that the absolute standardized residual values of compound nos. 53, 68 and 75 are larger than 3. These three compounds are recognized outliers after the application domain is defined. Then, the structure of application domain of the QSAR model was defined perfectly and the external validation of model could be evaluated using the remaining 19 compounds in the test set (*r_p_^2^* = 0.7656, *RMSP* = 0.14).

In addition, after the application domain of the model was well defined and three outliers were deleted from the test set, the results of the criteria proposed by Golbraikh and Tropsha [45] for the test set in the model are shown as follows, *k* = 0.9282 , *k'* = 1.0084 , (*R*^2^_o_ − *R*^2^)/*R*^2^ = –0.0115, (*R'*^2^_o_ − *R*^2^)/*R*^2^ = −0.0378. Obviously, *k* and *kʹ* are in the domain of 0.85 ≤ *k* ≤ 1.15 or 0.85 ≤ *k'* ≤ 1.15, and all values of *R*^2^, *R_o_*^2^, and *R_o_ʹ*^2^ nearly equal to 1. And (*R*^2^ − *R_o_*^2^)/*R*^2^ and (*R*^2^ − *R_o_*'^2^)/*R*^2^ are less than 0.1, all are in appropriate zone. These results confirm that the model has a good predictive power for an external sample, which indicate that the model can be used as a great predictive model for the placental barrier properties analysis of drug molecules after the application domain is defined.

There is a lot of literature on experimental determination of the placental barrier, but only four papers [27,28,46,47] mentioned high-throughput screening and the use of the QSAR method in studies on the placenta barrier. Giaginis *et al*. [28] created a PLS model with lower *r*^2^ and bigger *s* or *RMSE*. In our research, all compounds are directly derived from Giaginis’ data [28]. The method of PLS variable selection is employed to quickly select the optimal descriptor set from 620 DRAGON descriptors to build the PLS regression model. The data set is divided into 65 compounds in the training set and 19 components in the test set due to modeling and external validation. The statistics of the QSAR model in this study (*n* = 65, *A* = 7, *m* = 48, *r*^2^ = 0.9064, *RMSE* = 0.09, *q*^2^ = 0.7323; *n_p_* = 19, *r_p_^2^* = 0.7656, *RMSP* = 0.14) ensure our models’ strong competitiveness compared with the results of Giaginis models [28]. Using PLS method, a good quality PLS regression model can be quickly established for 48 optimal descriptors from 3764 descriptors and the CI values of compounds.

### 2.5. Mechanistic Interpretation

To infer whether the optimal descriptors were selected reasonably, the mechanistic interpretation of QSAR model was carried out according to the definition of descriptors. As Wold [45] suggested, because the descriptor VIP value is larger, this indicates this descriptor is significant for the PLS model. After model generation and validation, we interpret the selected descriptors that were used in the PLS models according to the most important VIP value. Therefore, seven descriptors (nHDon, TI2_L, P_VSA_p_2, nRNH2, ATSC1s, CATS2D_08_DA and SM1_Dz(p)) were considered to be the most significant descriptors according to the VIP values. The seven selected descriptors are functional group counts (nRNH2 and nHDon), P_VSA-like descriptors (P_VSA_p_2), 2D matrix-based descriptors (TI2_L and SM1_Dz(p)), 2D auto-correlations (ATSC1s), and CATS 2D descriptor (CATS2D_08_DA) [48].

Among the more important factors affecting the placental barrier permeability, it can be found that the number of donor atoms for H-bonds(N and O) (nHDon) and CATS2D Donor-Acceptor at lag 08 (CATS2D_08_DA) reflecting the polarity and hydrogen bonding capability of compounds are the most important descriptors. Then, nRNH2 refers to number of primary amines (aliphatic), P_VSA_p_2 refers to P_VSA-like on polarizability (bin 2) and SM1_Dz(p) refers to spectral moment of order 1 from Barysz matrix weighted by polarizability. These three descriptors indicated that molecular polarity and lipophilicity are important factors for permeability. TI2_L refers to second Mohar index from Laplace matrix. ATSC1s refers to Centred Broto-Moreau autocorrelation of lag 1 weighted by I-state. Also, the compounds’ lipophilicity made a considerable contribution in the transport of compounds across the human placenta. Our given analyses are consistent with the literature results [28] that compounds which possess a relatively high number of hydrogen bond acceptor or donor sites and thereupon low lipophilicity may exhibit reduced transport across the placental barrier [49]. Thus, the PLS regression model whose descriptors were chosen by PLS variable selection method is feasible in predicting the placental barrier permeability.

## 3. Experimental Section

**Figure 3:**
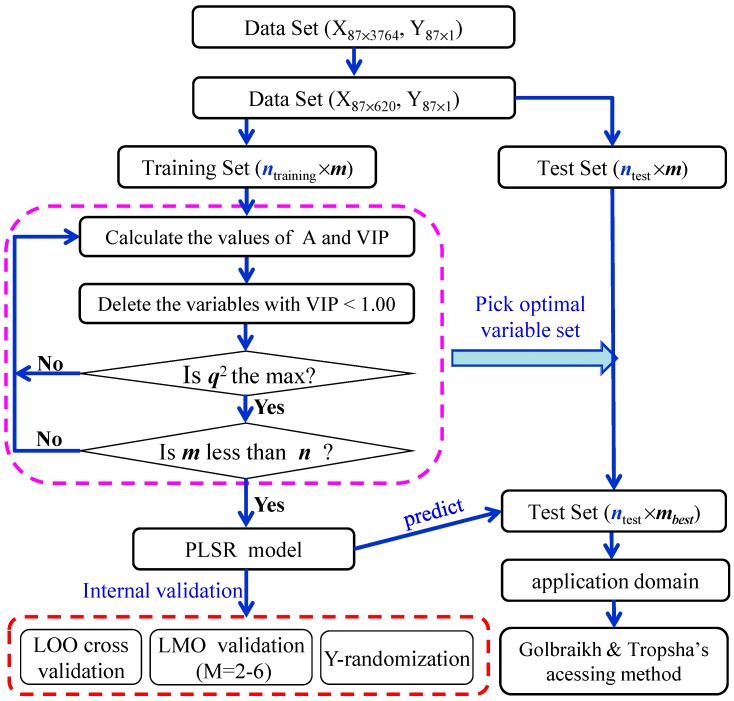
Sketch map for modeling and validation process of the CI value.

The procedure for developing the PLS models between the placental barrier permeability (CI) and molecular descriptors consists of data collection, descriptor calculation, variable selection, model development and validation, and application domain. The flow diagram of the procedure is shown in Figure 3.

### 3.1. Data Collection

**Figure 4:**
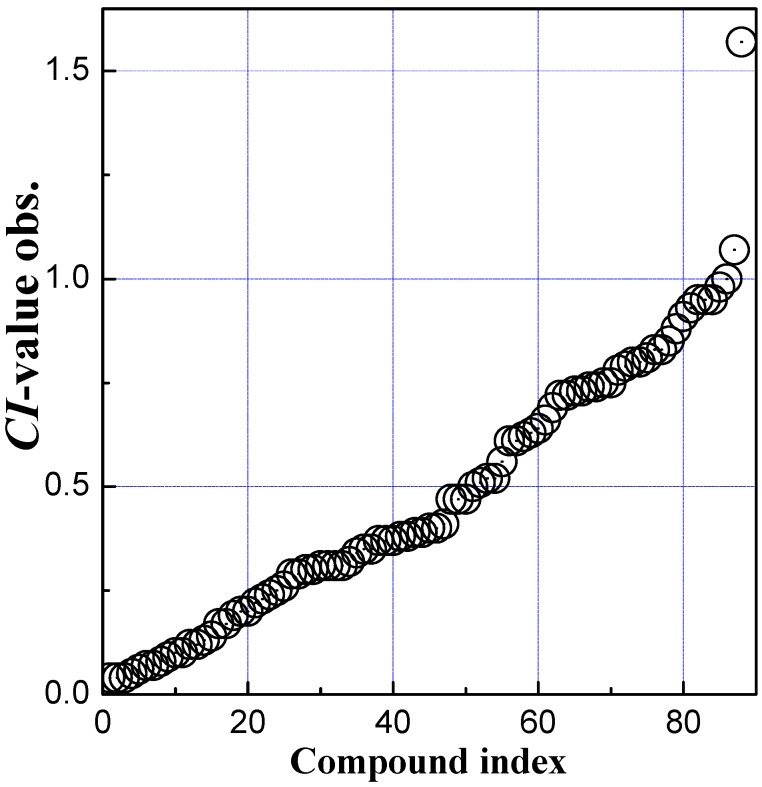
Distribution of the CI value observed of 88 drugs.

Eighty-eight compounds and their CI values are directly taken from the literature [28]. Here, CI = clearance of compound under study/clearance of a reference compound (antipyrine) [50,51,52]. The serial numbers, compound names and the experimental CI values of the compounds are listed in Table 3. The experimental CI values of 88 compounds are uniformly distributional (see Figure 4). From Figure 4, only one CI value is much bigger than others and the value is 1.57 (see mefloquine in Table 3), which indicates that the transport of mefloquine across the placenta exceed that of antipyrine. Mefloquine should be considered as an outlier and deleted from the data set. The other CI values are widespread and distributed in the range of 0.04–1.07. These compounds have diverse structures and belong to different drug genres, such as analgesic, antiviral, barbiturate, neuroleptic, and benzodiazepine, *etc.*

### 3.2. Descriptor Calculation and Pretreatment

Three thousand six hundred and seventy four molecular descriptors for each of 87 compounds were calculated by the Dragon software (version 6.0) [31]. The descriptors involve 19 categories, constitutional indices, ring descriptors, topological indices, walk and path counts, connectivity indices, information indices, 2D matrix-based descriptors, 2D autocorrelations, burden eigenvalues, P_VSA-like descriptors, ETA indices, edge adjacency indices, functional group counts, atom-centred fragments, atom-type E-state indices, CATS 2D, 2D atom pairs, molecular properties, and drug-like indices.

The values of one or many descriptors could be zero or a constant for all molecules due to the absence of some special atoms and these descriptors should be deleted. Furthermore, the descriptor with a standard deviation of <0.001 should be deleted due to little statistical meaning. If the correlation coefficient between two descriptors is greater than 0.90, then remove any one of the two descriptors. Then, the remaining 620 descriptors are obtained.

### 3.3. Variable Selection

The data set of 87 compounds is randomly divided into a training set of 65 samples and a test set of 22 ones. The PLS variable selection method [29] is selected to pick out optimal descriptors from the 620 descriptors in the training set. Taking the 620 descriptors as independent variable matrix (X) and CI values as dependent variable matrix (Y), the PLS variable selection and modeling are performed where the *q*^2^ obtained in the LOO cross-validation is taken as an objective function. Then, the variables with high VIP values (typically greater than 1) are extracted from X matrix as modeling variables [29,40,53]. The VIP value of the *j*th variable is defined as follows (Equation (1)) [40],

(1) $$VIP_{j}=sqr\left[m\times \sum_{a=1}^{A}{\omega^{2}}_{ja}\times r^{2}(y,t_{a})/\sum_{a=1}^{A}r^{2}(y,t_{a})\right]$$

where

(2) $$r^{2}(y,t_{a})=1-\sum_{i=1}^{n}{\left(y_{i}-t_{ia}\right)}^{2}/\sum_{i=1}^{n}{\left(y_{i}-\bar{y}\right)}^{2}$$

where *m* is the number of original variables, *ω is* a weight vector corresponding to the optimal latent variables (*A*), *r*(*y*, *t_a_*) is the correlation coefficient between Y vector and the score vector of the a^th^ latent variable (Equation (2)) [40].

### 3.4. Model Development and Validation

Taking a suitable latent variable number (*A*), a model between the CI values and optimal descriptors for 65 samples/drugs in training set is built by the PLS regression. The model is firstly internally validated by a LOO and LMO cross-validation. Here, the validated correlation coefficient (*q*^2^) (Equation (3)) is used to assess the quality of cross-validation [40,54,55,56].

(3) $$q^{2}=1-\frac{\sum_{i=1}^{n}{\left({\hat{y}}_{i}-y_{i}\right)}^{2}}{\sum_{i=1}^{n}{\left(y_{i}-\bar{y}\right)}^{2}}$$

where *y*_i_ and $\hat{y}$ are the *i*th experimental CI and that predicted by the LOO or LMO validation, respectively; $\bar{y}$ is the mean of CIs; *n* is the number of the samples in the training set.

Unlike the LOO, the LMO cross-validation randomly picks out many samples (M) rather than one each time and the remaining (n-M) samples in the training set are used to develop a model and then the model is employed to predict the CI values of the M samples. The procedure is repeated many times. In this study, M = 2, 3, 4, 5, and 6, respectively, and LMO cross-validation repeated 10 times. If a model has a high *q*^2^_LOO_ (>0.5) in LOO validation or high average *q*^2^_LMO_ (>0.5) in LMO validation, the obtained model could be thought robust.

Furthermore, the *y*-randomization test [43,45] is used to evaluate the possibility of chance correlation for a model. In this test, the dependent-variables (CI values) are firstly randomly shuffled, a model between the randomized CI values and the original independent-variables (descriptors) is developed [57]. In our study, the *y*-randomization test is repeated 10 times. If both the average values of *r*^2^s (*r*^2^*_Yrand_*) and *q*^2^_LOO_s (*q*^2^*_Yrand_*) obtained in *y*-randomization tests are low enough, it is indicated that the resulting model has no chance correlation. It is based on the following four criteria [43,45]: (i) *q*^2^*_Yrand_* < 0.2 and *r*^2^*_Yrand_* < 0.2, no chance correlation; (ii) any *q*^2^*_Yrand_* and 0.2 < *r*^2^*_Yrand_* < 0.3, negligible chance correlation; (iii) any *q*^2^*_Yrand_* and 0.3 < *r*^2^*_Yrand_* < 0.4, tolerable chance correlation; (iv) any *q*^2^*_Yrand_* and *r*^2^*_Yrand_* > 0.4, recognized chance correlation.

A good result in the internal validation (LOO and LMO) could ensure that the model is robust but not ensure that the model has high predictive power for an external sample. It is necessary to execute an external validation. In this study, the model developed in the training set of 65 samples was used to predict the CI value of 22 drugs in the test set. The predictive correlation coefficients (*r_p_^2^*) and root mean square error (*RMSP*) are used to evaluate the predictive power of the model.

Though high *r_p_*^2^ and low *RMSP* can interpret that the model is predictable, Golbraikh and Tropsha recommended the other statistical parameters to assess the model predictive ability [40,45]. The statistical parameters include (i) correlation coefficient *R* between the predicted and observed activities; (ii) coefficients of determination (predicted *versus* observed activities *R*_0_^2^, and observed *versus* predicted activities *R'*_0_^2^); (iii) slopes *k* and *k*' of the regression lines through the origin. They considered a model to be well predictable, if the following four conditions are satisfied: *Q*^2^ (the validated correlation coefficient) > 0.5; *R*^2^ > 0.6; $(R^{2}-R_{0}^{2})/R^{2}<0.1$ or $(R^{2}-R\prime_{0}^{2})/R^{2}<0.1$; 0.85 ≤ *k* ≤ 1.15 or 0.85 ≤ *k'* ≤ 1.15.

### 3.5. Application Domain

The application domain of a model is defined by a leverage [40,45], *h_i_* (Equation (4)).

(4) $$h_{i}=x_{i}^{T}{\left(X^{T}X\right)}^{-1}x_{i}\;(i=1\text{, …, }n)$$

where *x_i_* is the descriptor row vector of the *i*th compound; *X* is the *n* × *k* matrix of *k* descriptor values for *n* training set compounds, where *k* is the number of model variables, and *n* is the number of the samples in training set. The superscript “*T*” refers to the matrix/vector transpose. The control leverage h* is set as 3*k*/*n*.

## 4. Conclusions

An optimal descriptor set with 48 descriptors is rapidly derived from a large number of DRAGON descriptors according to descriptor VIP values by the PLS variable selection method. Then, a QSAR model based on an optimal descriptor set and the CI value of 65 compounds were built and used to predict the CI of 19 compounds with a well-defined application domain. The model presented excellent internal fitness and external prediction power by regression statistical parameters. The results of LOO and LMO cross-validation show the model is robust. The performance in *y*-randomization demonstrates the model does present acceptable chance correlation. The external prediction powers were evaluated as well as the criteria proposed by Golbraikh and Tropsha, and the results show the good statistical quality and predictive ability of the model. Therefore, it is expected that the QSAR model could be used to predict the placental barrier permeability of drug candidates with a well-defined application domain without experimental values.
